# Over-the-Scope Clip-Associated Endoscopic Muscular Dissection for Seven Cases of Small Gastric Submucosal Tumor: A Video-Based Case Series

**DOI:** 10.1155/2021/4578191

**Published:** 2021-03-19

**Authors:** Xin Li, Rongfen Wei, Jianfu Qin, Fei Qin, Peng Peng, Mengbin Qin, Shiquan Liu, Jiean Huang

**Affiliations:** Department of Gastroenterology, The Second Affiliated Hospital of Guangxi Medical University, Nanning, 530007 Guangxi Province, China

## Abstract

**Objectives:**

To evaluate the methodology, feasibility, safety, and efficacy of a novel method called over-the-scope clip- (OTSC-) associated endoscopic muscular dissection for small GSMT.

**Methods:**

A pilot study on small GSMT diameter ≤ 1 cm was performed. OTSC-associated endoscopic muscular dissection was based on the requirement of OTSC apparatus and ESD technique; after ligaturing the bottom of small GSMT by OTSC, ESD was performed to resect the tumors, and the wounds of ESD were closed by clips finally. All the patients were followed up for more than 3 months, and the complications during and after OTSC-associated endoscopic muscular dissection were recorded.

**Results:**

A total of 7 consecutive patients with small GSMT were included. All tumors were completely dissected without any perforation or infection during and after the procedure in all cases, while three patients had mild abdominal pain, and one experienced postoperative bleeding after the procedure which was treated by the endoscopy with titanium clips. All the patients were followed by endoscopy three months later, all the wounds healed well, and all the OTSCs were still in the gastric wall.

**Conclusions:**

OTSC-associated endoscopic muscular dissection as a novel endoscopic interventional therapy should be a convenient, safe, and effective therapy for small GSMT. The short-time outcome is excellent, whereas long-term effect is unclear, and the further follow-up is needed to schedule.

## 1. Introduction

Gastric submucosal tumor (GSMT) is one of the most common tumors in stomach; it usually originates from muscularis mucosa, submucosal layer, and muscularis propria layer, including stromal tumor, leiomyoma, lipoma, fibroma, and heterotopic pancreas, and some GSMTs may become malignant. We can know about their origin and size by endoscopic ultrasonography, but benign and malignant diagnosis depends on pathology. Small size cannot guarantee a specific malignant risk for gastric SMTs [1], to prevent recurrence, definitive diagnosis, and aggressive resection while the tumor size is 20 mm or less is recommended [2]. In the management of GSMT, therapeutic methods evolved from endoscopic mucosal resection (EMR), endoscopic submucosal dissection (ESD), and endoscopic full-thickness resection (EFTR) to laparoscopic surgery, depending on the origin, size, benign, and malignance. ESD is becoming the most common therapy for GSMT [3] and also widely used in the treatment for early carcinoma of digestive tract and precancerous lesion [4].

The main complications of ESD are perforation and bleeding, especially for the lesions from muscularis propria layer, and both of them could be effectively remedied by clips or endoscopic suture. However, there are still some risks when perforation occurs during ESD. Gastric juice and blood may flow into peritoneal cavity then cause peritonitis, serous hemorrhage is difficult to manage, and the tumor especially the small SMT may drop into the peritoneal cavity. So it is better to avoid perforation. Laparoscopic surgery can solve these problems, but the surgical injury is large, and it is not suitable for small SMT. As a result, prophylactic closure before ESD or EFTR is addressed and becomes more and more popular; it bases on OTSC, titanium clips, or endoscopic nylon rope suture. Prophylactic closure offers safety and high success rate for gastrointestinal tumors and residual neoplastic tissue [5, 6]. Up to now, there is no written report on OTSC prophylactic closure for small GSMT yet. Therefore, in order to establish a safer and more effective therapy, we address a novel treatment for small GSMT called over-the-scope clip- (OTSC-) associated endoscopic muscular dissection; this article presents our pilot study on the methodology, feasibility, safety, and clinical findings using OTSC-associated endoscopic muscular dissection for small GSMT.

## 2. Materials and Methods

### 2.1. Patient Inclusion and Exclusion Criteria

This observational study was carried out in the Second Affiliated Hospital of Guangxi Medical University from July 2017 to March 2020. Eligible patients of GSMT originated from the muscularis propria layer and diameter ≤ 1 cm (the maximum diameter of OTSC is 11 mm) were included in the study. All the patients accepted the interventional procedures for small GSMT voluntarily, and all included cases for analysis were followed up for at least 3 months.

Tumors originated from mucosa layer, submucosa layer, and diameter > 1 cm were excluded. Severe cardiopulmonary failure, coagulation disorders, and thrombocytopenia patients were also excluded.

This study was approved by the Medical Ethics Committee of the Second Affiliated Hospital of Guangxi Medical University on May 18, 2017, approval number: 2017(KY-E-0162). Written informed consent was given to all patients.

### 2.2. Concept and Methods

**Figure 1:**
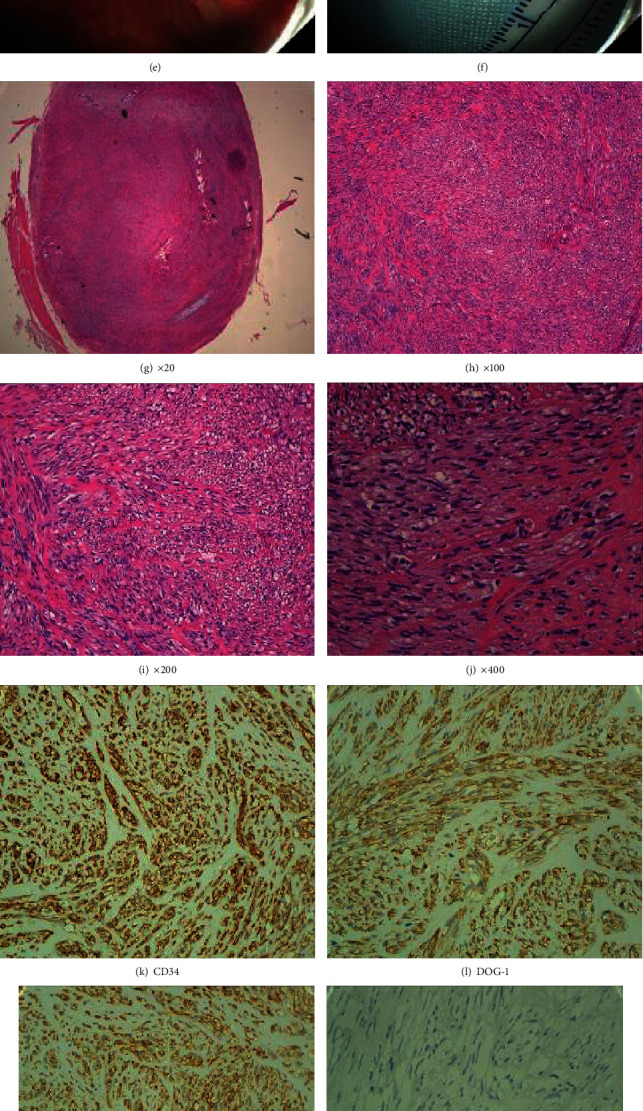
Procedure of OTSC-associated endoscopic muscular dissection for the dissection of small gastric submucosal tumor. (a) A small gastric submucosal tumor of fundus; (b) the tumor was completely absorbed into the transparent cap of OTSC apparatus; (c) the bottom of tumor was ligatured by OTSC; (d) dissection of the tumor; (e) the wound was clipped after the procedure; (f) the resected tumor; (g–j) HE staining, magnified pathological images; (k–q) immunohistochemical staining, CD34(+), DOG-1(+), CD117(+), SMA(-), S-100(-), desmin(-), and Ki-67(+, <1%).

The procedure was performed by a skilled endoscopist with hundreds of ESD experience. The tumor was marked by electrocoagulation first, then completely absorbed into the transparent cap (the endoscopic view was full of red) of OTSC apparatus; the OTSC was released to ligature the bottom of the tumor at last. Mucosal layer, submucosal layer, and muscularis propria layer were incised successively by dual knife or IT knife after ligation; sometimes, submucosal injection (normal saline plus methylene blue) was necessary for incision, and the tumor was dissected finally. During the procedure, hot biopsy forceps electrocoagulation was used to stop the bleeding when necessary and titanium clips were used to close the wound (see Figures 1(a)–1(f) and Video clip 1 in the Supplementary Material for deep understanding).

All the patients were required to rest in bed on the first night after the procedure, and fast for three days, proton pump inhibitor was prescribed for 6-8 weeks. Antibiotics were used to prevent infection for all individuals in this study. The complications were recorded during and 3 months after the procedure, the symptom of abdominal pain, hematemesis, melena, and fever and the sign of pneumoperitoneum and peritonitis were observed for perforation, infection, and postoperative bleeding. All the patients were reexamined through gastric endoscopy three months after the OTSC-associated endoscopic muscular dissection for observation of the wounds.

The definitive diagnosis of GSMT depends on the pathological results. Before OTSC-associated endoscopic muscular dissection, EUS was used to determine that the tumors originated from muscularis propria. Because the tumors are small, it is difficult to operate EUS-FNA, and the integrity of the tumors needs to be ensured, so EUS-FNA was not performed before OTSC-associated endoscopic muscular dissection.

## 3. Results

### 3.1. Patient Characteristics

Table 1 shows the characteristics of the small GSMT patients, including gender, age, tumor diameter, location, pathology, complications, and operation duration. A total of 7 patients with OTSC-associated endoscopic muscular dissection were included for the analysis in this study. The data were unsuitable for statistical analysis because the number of cases was too small (see Dataset 1 in the Supplementary Material for raw data).

### 3.2. Clinical Findings

Figure 1 and Video 1 show the steps of OTSC-associated endoscopic muscular dissection for dissection of small GSMT. Compared with conventional ESD technique, it was easier to resect the tumor by OTSC-associated endoscopic muscular dissection, because the tumor became fixed and protruded. All the seven patients' tumors were successfully resected through OTSC-associated endoscopic muscular dissection, and no perforations or severe bleeding was observed during the procedure. The operation duration was 20 minutes averagely. The patients were required to stay in hospital for 3 to 5 days after the procedure for safety considerations in this study. Three patients had mild abdominal pain for one to two days, and one patient experienced postoperative bleeding one week after the procedure with the symptom of melena. The bleeding was confirmed as the result of an exposed blood vessel and finally stopped through the endoscopic treatment with clips. No hematemesis, fever, pneumoperitoneum, and peritonitis were observed in all patients.

The pathological results of 7 cases were all stromal tumors with low-grade malignancy. HE staining showed spindle cell tumors; immunohistochemical staining showed CD34(+), DOG-1(+), CD117(+), SMA(-), S-100(-), desmin(-), and all cases' Ki-67(+, <1%) (see Figures 1(g)–1(q)). None of them needed chemotherapy or surgical operation. All patients could return to normal activities after they were discharged from hospital. One hundred percent of patients were satisfied with this novel procedure. The endoscopy was performed for every patient three months after the procedure, all the wounds healed well, and all the OTSCs were still in the gastric wall, without any discomforts.

## 4. Discussion

With the improvement of life quality and healthy consciousness, more and more people require to resect the small GSMT. ESD is used for treating early carcinoma of digestive tract at first, along with the expansion of indications; now it is becoming the most common therapeutic method for GSMT [7]. OTSC is usually used for gastrointestinal bleeding, fistula, and endoscopic iatrogenic perforation [8–14] and also used for closing anastomotic leakage after colorectal cancer surgery [15] and entry site closure in POEM and G-POEM [16]. Recently, OTSC is reported using for prophylactic closure in ESD or EFTR in order to make the procedure safer and more effective [5, 6], as well as endoscopic suture [17].

One of the main complications of ESD is perforation, which is at risk ranging from 1.2% to 9.7% [18, 19]. Because titanium clips and endoscopic suture could effectively close the perforation, it is not frightening anymore. However, in most of endoscopists' opinion, perforations should be avoided, and preloading ligation of OTSC before ESD was designed for small GSMT resection. Because of the preloading ligation to the bottom of the tumor with OTSC, it is nearly impossible to punch a hole to peritoneal cavity during ESD or even during EFTR. In this study, all tumors were completely resected by OTSC-assisted ESD, no perforations occurred during or after the procedure, no tumors dropped into the peritoneal cavity, and no patient developed peritoneal infection. It demonstrates that OTSC prophylactic closure before ESD can effectively avoid the above adverse events and make the procedure safer.

Bleeding is another common complication of ESD; the risk is ranging from 0.6% to 15.6% [15, 16]. In our study, there was no severe bleeding during the procedure, one patient experienced postoperative bleeding, and the bleeding was cured through endoscopic treatment of clips. This adverse event was finally confirmed as the result of an exposed blood vessel. OTSC is an effective hemostasis therapy for nonvariceal upper gastrointestinal bleeding, but it also has 8% rebleeding rate [10]. It demonstrates that the OTSC cannot block blood flow completely. So we have to highlight that the wound must be electrocoagulated in time and tightly closed with titanium clips when the OTSC-associated endoscopic muscular dissection procedure was finished.

As we know, vision clarity is the main influencing factor for gastrointestinal tumor resection [20, 21]. We found that OTSC prophylactic closure can keep a clear procedure vision and make the small GSMTs easy to be resected. First, no air or just a little air enters the peritoneal cavity, and enough air in the gastric cavity may keep a clear vision. Secondly, the OTSC can further fix and protrude the tumors, which is easier to be dissected. Thirdly, there are no obvious bleeding during procedure. As a result, the endoscopist may spend less time on the ESD procedure. In this study, the procedure duration is 20 minutes averagely.

Stromal tumor is the most common type of gastrointestinal submucosal tumors originated from muscularis propria layer; it was once considered as a low malignant potential tumor when it is smaller than 2 centimeters. But recent literature reported that the rate of invasion and metastasis reached 11.4% in 378 cases of small gastrointestinal stromal tumor (GIST) when they were diagnosed at the first time [22]. It is suggested to observe closely by some scholars, but doctors can only observe the size of tumors and then produce a procedure passively when they become lager [23, 24]. So it is important to assess the invasion and metastasis risk of small GIST. First, different parts of small GISTs have different characteristics, small gastric stromal tumors show low malignant risk except tumors from corpus ventriculi, and tumors from other parts of gastrointestinal especially from duodenum and small intestinal usually show high invasiveness risk. Second, endoscopic ultrasonography is another method to assess the malignant risk of small GIST, and the high-risk factors include irregular border, cystic change, strong echo, or irregular echo [25–27]. The above two cases are suitable for OTSC-associated endoscopic muscular dissection treatment when the diameter is ≤1 cm, so small gastrointestinal submucosal tumors (≤1 cm) need to be diagnostically resected. Till now, there is little data on the time of free survival of small gastrointestinal submucosal tumors. Current literature reports that the recurrence and metastasis risk of small gastric stromal tumors is zero regardless of mitotic count, and the risks are zero and 50%-54% in nongastric origin stromal tumors when the mitotic counts are ≤5/5 mm^2^ and > 5/5 mm^2^, respectively [28]. Little literature reports the average time of recurrence of small gastrointestinal submucosal tumors after resection. In our study, no recurrence has been observed during follow-up yet.

There is a similar method of resection by snaring after using OTSC, which has been reported as “EMR with an over-the-scope clip (EMRO)”. EMRO can be considered a safe and effective treatment modality for relatively small (≤10 mm in diameter) superficial nonampullary duodenal epithelial tumors (SNADETs) with fibrosis [29]. Both EMRO and OTSC-associated endoscopic muscular dissection can greatly reduce the risk of bleeding and perforation. Compared with EMRO, the advantages of OTSC-associated endoscopic muscular dissection are as follows: first, OTSC-associated endoscopic muscular dissection can ensure the complete resection of the muscularis propria tumor; second, OTSC-associated endoscopic muscular dissection only resects the muscularis propria tumor and preserves the mucosa, which can shorten the healing time. Because the sample size is too small, the comparison between the two methods needs more data and research.

In conclusion, OTSC-associated endoscopic muscular dissection is an innovational, effective, safe, and convenient method for small GSMT. It has an advantage over EFTR in shorter operation duration and less peritonitis rate, while both of them show the same treatment success rate. The side effect of OTSC remained in gastric wall needs further follow-up observation. However, there were some limitations in this study. The sample size of this pilot study was small, and a larger one based on these preliminary results needs to be carried out. This was not a controlled study with the comparison of other conventional interventional therapies; therefore, a rigorous randomized clinical trial should be designed to provide more evidence for the practice of OTSC-associated endoscopic muscular dissection.

## 5. Conclusions

OTSC-associated endoscopic muscular dissection as a novel endoscopic interventional therapy should be a convenient, safe, and effective therapy for small GSMT. The short-time outcome is excellent, whereas long-term effect is unclear, and the further follow-up is needed to schedule.

## Figures and Tables

**Table 1:** Patient demographics and clinical results.

Case	Gender	Age	Tumor diameter	Location	Pathology	Complications	Operation duration
1	F	43 y	5 mm	Fundus	Stromal tumor	None	20 min
2	F	54 y	7 mm	Fundus	Stromal tumor	Postoperation bleeding	28 min
3	F	48 y	7 mm	Fundus	Stromal tumor	None	20 min
4	F	53 y	8 mm	Fundus	Stromal tumor	None	15 min
5	F	65 y	10 mm	Fundus	Stromal tumor	None	42 min
6	F	55 y	8 mm	Fundus	Stromal tumor	None	25 min
7	F	62 y	10 mm	Fundus	Stromal tumor	None	20 min

## Data Availability

Authors may provide the data in the Supplementary Information files that they submit alongside the manuscript.

## Supplementary Materials

Supplementary Materials Video clip 1: to show the process of OTSC-associated endoscopic muscular dissection. Dataset 1: to show the raw data of the small GSMT patients, including gender, age, tumor diameter, location, pathology, complications, operation duration, and treatment success rate.
